# QLFDGWO: Q-Learning-Guided Weighted Fitness–Distance Grey Wolf Optimizer for UAV Path Planning

**DOI:** 10.3390/biomimetics11060428

**Published:** 2026-06-15

**Authors:** Chen Huang, Beining Yang, Yan Huo

**Affiliations:** 1 College of Civil Aviation, Shenyang Aerospace University, Shenyang 110136, China; 2 College of Intelligent Science and Information Engineering, Shenyang University, Shenyang 110044, China

**Keywords:** grey wolf optimization, Q-learning, cosine nonlinear, UAV path planning

## Abstract

Traditional grey wolf optimizer (GWO) frequently suffers from insufficient search diversity, unstable stage transition, and premature convergence when addressing complex optimization tasks. To overcome these limitations, this paper proposes an improved grey wolf optimizer with a Q-learning-guided fitness–distance-weighted selector. For the proposed QLFDGWO framework, first, chaotic mapping is introduced to generate a more diverse initial population. A cosine nonlinear convergence factor is employed to improve adjustment capability during the search process. Additionally, a Q-learning-based strategy selection mechanism is constructed to enable adaptive switching between exploration and exploitation. To further improve the leadership structure of GWO, a Q-learning-guided fitness–distance-weighted selection mechanism is designed, in which the beta and delta wolves are selected by jointly considering fitness quality and spatial distance from the alpha wolf. A dynamic threshold-weighted update strategy is designed to enhance the convergence accuracy and stability of the population. Finally, the proposed algorithm is benchmarked against five representative optimization algorithms using the CEC2017 benchmark function set. Experimental results indicate that QLFDGWO achieves satisfactory performance in terms of optimization accuracy, convergence speed, and robustness. In addition, QLFDGWO is applied to three-dimensional (3D) unmanned aerial vehicle (UAV) path planning under a range of complex scenarios. Simulation results demonstrate that the proposed method can generate feasible, safe flight paths that satisfy terrain and obstacle constraints.

## 1. Introduction

Complex optimization problems are ubiquitous across scientific research and engineering practice [1,2,3,4], with representative application domains including control system design [5], industrial scheduling [6], power system optimization [7] and path planning [8]. For instance, Jo et al. [9] developed a Bayesian optimization-based large neighbourhood search method for large-scale electric vehicle charging station deployment. Yoon et al. [10] proposed a collaborative truck-and-robot method for urban service management under heat-wave conditions. It is well known that the swarm intelligence optimization algorithm simulates the collaborative behaviour and information sharing mechanism of biological groups in nature, demonstrating excellent advantages in global search ability, algorithm structure simplicity, and problem adaptability [11]. Typical algorithms include Particle Swarm Optimization (PSO) [12], Ant Colony Optimization (ACO) [13], and the Artificial Bee Colony Algorithm [14], etc. These algorithms have the advantages of simple structure and less tendency to fall into local optima [15,16]. The grey wolf optimizer (GWO), as a commonly used optimization algorithm, completed the optimization search by simulating the hierarchical structure and hunting behaviour of grey wolf groups [15]. This algorithm is characterized by a simple structure, few control parameters, and relatively fast convergence, and shows strong competitiveness in continuous optimization problems. However, when solving complex functions or multi-peak problems, the traditional GWO still has the following shortcomings: first, the linear decreasing mechanism of the convergence factor leads to an insufficient smooth transition between exploration and exploitation stages, affecting the search efficiency; second, individual update depends on the average of the top three individuals, which easily leads to a decrease in population diversity; and third, the algorithm is prone to fall into local optima in complex high-dimensional problems. In addition, the conventional GWO selects alpha, beta, and delta wolves mainly according to fitness ranking. When the leading wolves are located close to each other, the guidance information may become highly similar, which further reduces population diversity and increases the risk of premature convergence.

Recent studies on GWO improvement mainly focus on search balance, parameter adaptation, and population initialization [17,18,19,20]. In terms of improving the convergence mechanism, Zhang et al. [21] incorporated a dual-mode learning mechanism into GWO to improve the coordination between global and local searches, and also introduced a reflection mechanism, combining self-reinforcement, elite guidance strategies, etc., to reduce the risk of premature convergence. Chen et al. [22] proposed a balanced grey wolf optimizer (BGWO) for training backpropagation neural networks (BPNNs), and enhanced the coordination between exploitation and exploration in the original GWO. In terms of overcoming high-dimensional complex problems, Tian et al. [23] proposed a hybrid adaptive differential evolution grey wolf optimizer, which has excellent computational capabilities in high-dimensional problems. In terms of enhancing the global search ability, Zhu et al. [24] proposed a multi-layer perceptron (MLP) proxy model-based algorithm and an improved grey wolf optimizer with SPM chaotic mapping initialization, convergence coefficient updating and association learning. In terms of enhancing global optimization, Gupta et al. [25] expanded the search range of GWO by introducing the Levy flight strategy. To overcome the limitations of the algorithm’s local optimality, Rodrigues [26] created a GWO based on chaos theory. Cui et al. [27] enhanced the search ability of GWO by introducing a random perturbation strategy.

However, the GWO algorithm still has certain limitations in some aspects: Firstly, most of the improved GWO algorithms lack dynamic strategy adjustment mechanisms based on environmental feedback, and there is still room for improvement in their adaptive control capabilities for different search stages. Secondly, the performance of the algorithm in all dimensions should be tested and analyzed, and the stability of the algorithm should also be taken into account. Thirdly, in the standard GWO, beta and delta wolves are directly selected according to fitness ranking, which may weaken the diversity of leader guidance when the top-ranked individuals are concentrated in a similar region. Reinforcement learning, as a decision optimization method based on environmental feedback, has made significant progress in the fields of dynamic strategy selection and intelligent control. In recent years, reinforcement learning has gradually been introduced into swarm intelligence optimization algorithms to achieve dynamic selection and adaptive adjustment of search strategies. Niculescu-Faida et al. [28] combined the particle swarm algorithm with reinforcement learning to adaptively adjust the parameters of the PID controller, thereby conducting research on the automatic regulation and optimization system for reservoir water levels. The reinforcement learning mechanism provides an adaptive decision-making framework for swarm intelligence algorithms, which helps improve optimization accuracy, convergence stability, and resistance to premature convergence.

This paper presents an improved grey wolf optimization algorithm based on Q-learning-guided strategy selection and fitness–distance-weighted leader selection. The construction of Q-learning aims to achieve adaptive switching between exploration and exploitation; the proposed method can adaptively select the operator that can maximize the population state based on the current state. The main contributions are as follows:

(1) A Q-learning-guided fitness–distance-weighted leader selection mechanism is proposed, where alpha remains the best individual, and beta and delta are adaptively selected from high-quality candidates by balancing fitness quality and spatial diversity.
(2) A dynamic threshold-weighted update strategy is designed to adaptively adjust elite guidance weights based on population fitness differences, achieving a trade-off between convergence accuracy and population diversity.
(3) An improved grey wolf optimizer, integrating Tent chaotic initialization and a cosine nonlinear convergence factor, is used to optimize the initial population distribution and balances the exploration–exploitation transition, thus enhancing global search capability and convergence stability.
(4) The proposed algorithm is applied to three-dimensional (3D) unmanned aerial vehicle (UAV) path planning problems, and is verified in various complex terrain scenarios.

The remainder of the paper is organized as follows. In Section 2, the traditional GWO is briefly introduced. Section 3 elucidates the proposed QLFDGWO algorithm in detail. The experimental results of CEC2017 standard test functions are given in Section 4. Section 5 describes three-dimensional path planning of QLFDGWO. Finally, Section 6 summarizes this study and suggests future work.

## 2. Grey Wolf Optimizer

GWO is bio-inspired by the hunting behaviour and social hierarchy of grey wolves, in which the population is organized into four hierarchical levels: the alpha wolf (α), the beta wolf (β), the delta wolf (δ), and the ordinary members (ω). Specifically, α, β, and δ represent the individuals with the best, second-best, and third-best fitness values in the current iteration, respectively. Their positions are used to guide the search direction of the population; the remaining individuals are classified as ω-type members, and their positions are updated by following the top three individuals. This hierarchical mechanism ensures the dominant role of high-quality solutions in the evolution of the population. During the search process, GWO achieves optimization by simulating the behaviour of “surrounding–approaching–attacking” the prey. First, the population surrounds the current optimal solution; then, the search range is gradually narrowed through position updates; finally, the optimal solution is approximated in the convergence stage. The movement of ω wolves is mainly directed by α, β, and δ, allowing the population to search globally while gradually exploiting promising regions. As the iterations proceed, the control parameters gradually decrease, thus enabling the search process to transition from global exploration to local exploitation. The position update rule is formalized as

(1) $$X_{i}(t+1)=\frac{1}{3}(X_{1}+X_{2}+X_{3})$$

where $X_{i}(t+1)$ denotes the updated position of the *i*-th grey wolf at iteration *t* + 1. $X_{1}$, $X_{2}$ and $X_{3}$ represent the three candidate positions generated under the guidance of the leader wolves, which can be calculated by

(2) $$\left\{\begin{array}{l} X_{1}=X_{\alpha }-A_{1}D_{\alpha }\\ X_{2}=X_{\beta }-A_{2}D_{\beta }\\ X_{3}=X_{\delta }-A_{3}D_{\delta } \end{array}\right.$$

where $X_{\alpha }$, $X_{\beta }$ and $X_{\delta }$ are the position vectors of the *α*, *β*, and *δ* wolves, which are determined by the top three fitness rankings in the current population. $A_{1}$, $A_{2}$ and $A_{3}$ are coefficient vectors used to control the search step during position updating. $D_{\alpha }$, $D_{\beta }$ and $D_{\delta }$ are used to measure the distance vectors between the current wolf and the three leader wolves.

## 3. Proposed Method

### 3.1. Chaos Initialization

In the traditional GWO method, the positions of the initial population are randomly generated. However, this initialization approach often causes the population to concentrate in certain areas of the solution space, which may degrade the global search capability and increase the risk of convergence to suboptimal local solutions. To mitigate the drawback, chaotic mapping is adopted for population initialization to improve individual diversity and expand the search coverage.

Specifically, the Tent map, as a simple chaotic mapping, is used to generate initial population positions to boost population diversity. The position of each wolf is generated from a chaotic sequence, which is then mapped to the feasible solution space of the target optimization problem. The Tent map is expressed as

(3) $$X_{k+1}=\left\{\begin{array}{l} \mu X_{k},X_{k}<0.5\\ \mu (1-X_{k}),X_{k}\geq 0.5 \end{array}\right.$$

where $X_{k}$ denotes the chaotic variable generated by the Tent map at the *k*-th iteration, satisfying 0 < $X_{k}$ < 1, and *μ* = 1.99 is used to generate chaotic sequences and map them into the feasible domain of the decision variables.

The chaotic mapping can generate a set of highly random sequences, which are used to determine the initial position of each wolf. Different from the traditional random generation method, chaotic mapping can ensure that the population is uniformly distributed in the solution space, avoiding the excessive concentration of the initial population, thereby improving the global search ability.

### 3.2. Convergence Factor

In the GWO framework, α serves as a key control parameter for coordinating global search and local refinement. Traditionally, α usually decreases linearly. Although this strategy is simple, it has two main limitations. First, the transition from global exploration to local exploitation is too abrupt. The search state is determined by the absolute value of the coefficient vector $A_{i}$, where |$A_{i}$| > 1 indicates exploration and |$A_{i}$| < 1 indicates exploitation. Specifically, a larger |$A_{i}$| encourages the wolves to explore a wider search region, while a smaller |$A_{i}$| guides the wolves to approach the current leader positions for local exploitation; the linear decrease in α causes the range of |$A_{i}$| to shrink uniformly. As a result, the population tends to switch to the exploitation phase within a relatively short period, rather than through a smooth transition, which may prevent sufficient exploration of the global search space. Second, the search capability in the middle stage declines too quickly. Because the linear strategy reduces α at a fixed rate from the beginning, population diversity decreases continuously during the search process. This makes the algorithm more likely to converge prematurely and become trapped in local optima, especially in high-dimensional optimization problems.

To address the aforementioned issues, a nonlinear convergence factor adjustment mechanism based on the cosine function is proposed. In QLFDGWO, the convergence factor is defined as follows:

(4) $$a(t)=2\cos(\frac{\pi t}{2T_{\max}})$$

where *t* is the iteration counter and *T*_max_ is the preset maximum iteration number.

In the early stage of iteration when *t* is small, this function shows a slow downward trend. Each individual can have more time for global exploration, extending the entire exploration stage and enabling more comprehensive coverage of the solution search space. As the cosine function is a smooth curve, in the middle stage of iteration, the algorithm will gradually progress from the exploration stage to the development stage, achieving a smooth transition in the middle period and more stable group behaviour. As the iteration number *t* approaches *T*_max_, the function drops faster, which enhances the algorithm’s local search ability, thereby improving convergence precision and reducing convergence runtime. The introduction of this cosine-type nonlinear convergence factor achieves comprehensive exploration in the early stage, smooth transition in the middle stage and rapid convergence in the later stage, to solve high-dimensional complex optimization problems.

### 3.3. Q-Learning-Based Adaptive Strategy and Leader Selection

**Figure 1:**
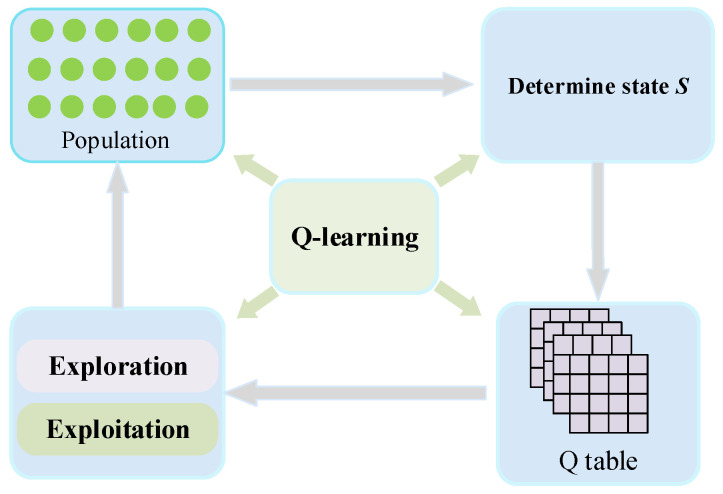
Q-learning-assisted operator selection strategy.

The position update rule in the GWO algorithm relies on the position information of three leading wolves (α,β,δ). However, this method assumes that the guiding roles of the three leading wolves in the optimization process are the same for all other wolves, and the update process is fixed, lacking flexibility. When dealing with complex multi-peak problems or high-dimensional problems, this static strategy is prone to causing early convergence or getting stuck in local optimal solutions. Therefore, Q-learning-based operator selection strategy is introduced to enhance the search capabilities of GWO. Its strategy is shown in Figure 1. The Q-learning-assisted operator selection strategy mainly includes Q-learning, state and action space, a reward function and a location update strategy.

#### 3.3.1. Q-Learning

Q-learning is a value-based reinforcement learning method. It gradually optimizes the strategy by learning from the feedback of the environment and selects the optimal action. By continuously updating the estimated return of different actions under each state, Q-learning guides the agent toward a better action–selection policy. The update rule is given by the following:

(5) $$Q(s_{t,}a_{t})=Q(s_{t,}a_{t})+\lambda (R_{t+1}+\gamma \underset{a}{\max}Q(s_{t+1,}a)-Q(s_{t,}a_{t}))$$

where $Q(s_{t,}a_{t})$ is the expected reward value for taking action in state $s_{t,}$. $R_{t+1}$ is the immediate reward obtained by the system after executing action $a_{t}$. γ controls the contribution of future rewards, and λ regulates how strongly the newly obtained information affects the current Q-value. γ = 0.8, and the learning rate adopts a decreasing form:

(6) $$\lambda (t)=\lambda_{0}(1-\frac{t}{T_{\max}})$$

This mechanism allows the algorithm to learn quickly at the beginning of the search and become more stable as the iteration proceeds. In Q-learning, the system takes actions based on the current state, and adjusts its future behaviour through reward feedback. Through this adaptive strategy update, Q-learning can flexibly switch between exploration and exploitation, enhancing the adaptive ability of the grey wolf optimization algorithm.

#### 3.3.2. State and Action Space

In QLFDGWO, the position and fitness of each wolf can be regarded as its state, and the action of each wolf is defined as the selection between the exploration strategy and the exploitation strategy. In the traditional GWO, the coefficient vector *A* is an important control parameter in the position updating process. It is calculated by the convergence factor and random variables, and is utilized to adjust the search direction and step size of grey wolves. Therefore, the magnitude of A directly reflects the current search tendency of the population. When |*A*| > 1, the wolves demonstrate divergent movement relative to the leader wolves to conduct global exploration; when |*A*| < 1, the wolves demonstrate convergent movement toward the leader wolves to conduct local exploitation. Therefore, the current search state is defined as follows:

(7) $$S_{t}=\left\{\begin{array}{l} 1,\;if\;\frac{1}{3}\sum_{k=1}^{3}\left|A_{k}\right|\leq 1\\ 0,\;\mathrm{otherwise} \end{array}\right.$$

The current environmental state is determined by the average amplitude of three input vectors. The state space is a discrete set: $S=\left\{0,1\right\}$; 0 represents exploring the environment, and 1 represents exploiting the environment.

The action space is *A* = {0, 1}. Action 0 executes the exploration strategy; action 1 executes the exploitation strategy. Additionally, a *ε*-greedy strategy is adopted for action selection:

(8) $$a_{t}=\left\{\begin{array}{l} a\sim U(A),\;\mathrm{with}\;\mathrm{probability}\;\varepsilon \\ \arg\max Q(S_{t},a),\;\mathrm{with}\;\mathrm{probability}\;1-\varepsilon \end{array}\right.$$

where $a_{t}$ denotes the action selected at time *t*, and ε is the exploration probability, which is set to 0.1 in this study. With probability ε, the algorithm randomly selects an action from *A*; with probability 1−ε, it selects the action with the maximum Q-value under the current state $S_{t}$.

#### 3.3.3. Reward Function

The reward for each action is determined by the change in fitness. When the fitness of the new solution is better than that of the old solution, a positive reward of +1 is given; otherwise, a negative reward of −1 is given to guide the update of the Q-table. Specifically, the reward function can be defined as follows:

(9) $$R_{t}=\left\{\begin{array}{l} +1,\;f_{new}<f_{old}\\ -1,\;f_{new}\geq f_{old} \end{array}\right.$$

where $f_{new}$ denotes the fitness value of the new solution after position updating, and $f_{old}$ denotes the fitness value of the original solution before position updating.

#### 3.3.4. Q-Learning-Guided Fitness–Distance-Weighted Leader Selection

In the proposed mechanism, the leadership hierarchy is constructed by combining elite guidance with distribution diversity rather than relying only on fitness ranking. This enables the selected leaders to provide more complementary search information and helps alleviate premature convergence.

Alpha is still selected as the best individual, while beta and delta are selected from the top-ranked candidate individuals according to a fitness–distance-weighted score. The candidate pool is composed of the top 30% individuals in the current population. The *i*-th candidate is calculated as follows:

(10) $$S_{f(i)}=1-(f_{i}-f_{\min})/(f_{\max}-f_{\min}+\varepsilon )$$

where $f_{i}$ represents the fitness value of the *i*-th candidate, $f_{\min}$ and $f_{\max}$ are the minimum and maximum fitness values in the candidate pool, respectively, and ε is a small constant used to avoid division by zero.

To measure the spatial diversity of the candidate leader, the normalized distance between the candidate individual and alpha is calculated as follows:

(11) $$d_{i}={\left\|X_{i}-X_{\alpha }\right\|}^{2}$$

(12) $$S_{d(i)}=d_{i}/(d_{\max}+\varepsilon )$$

where $X_{i}$ is the normalized position of the *i*-th candidate, $X_{\alpha }$ is the normalized position of alpha, $d_{i}$ is the distance between the candidate and alpha, and $d_{\max}$ is the maximum distance among all candidates. A larger $S_{d(i)}$ indicates that the candidate can provide more diverse guidance information.

The final fitness–distance-weighted score $FW_{i}$ is defined as follows:

(13) $$FW_{i}=\omega \cdot S_{f(i)}+(1-\omega )\cdot S_{d(i)}$$

where ω is the weight coefficient used to balance fitness quality and spatial diversity. When ω is large, the selection process pays more attention to fitness quality. When ω is small, spatial diversity has a greater influence on the selection result.

To avoid using a fixed weight, Q-learning is used to adaptively select ω from the candidate set Ω = {0.70, 0.80, 0.90, 0.98}.

After calculating $FW_{i}$, the candidates with the highest two scores are selected as beta and delta wolves. Through this mechanism, the leader wolves can maintain good fitness quality while providing more diverse search guidance, thereby improving the balance between convergence accuracy and population diversity.

#### 3.3.5. Location Update Strategy

To maintain an effective trade-off between global search and local refinement, different location update strategies are adopted at different search stages. At the initial stage, the algorithm emphasizes population diversity and search range expansion to avoid premature convergence. During the final stage of optimization, it focuses on exploiting promising regions and improving convergence accuracy. This stage-dependent design enhances both the global search capability and the convergence stability of the algorithm.

(1) Exploration phase

In the exploration phase, to enhance population diversity and improve the overall search capability, a random differential update mechanism is employed to improve population diversity and expand the search range. Specifically, when performing action 0, the random differential update mechanism is adopted:

(14) $$X_{i}^{t+1}=X_{i}^{t}+r⊙(X_{r_{1}}^{t}-X_{r_{2}}^{t})$$

where $X_{i}^{t}$ denotes the position of the *i*-th grey wolf at iteration *t*, and $X_{i}^{t+1}$ denotes the updated position of the *i*-th grey wolf at iteration *t* + 1. $r_{1}$ and $r_{2}$ are randomly selected individuals; *r* is in the range (0, 1).

(2) Exploitation stage

In the exploitation stage, a dynamic threshold is introduced to adaptively choose the update strategy. In Equation (15), *g* is an adjustment coefficient that controls the sensitivity of the threshold, and *a*(*t*) is the nonlinear convergence factor. A larger g strengthens the fitness-weighted guidance, while a smaller *g* helps preserve population diversity. When Equation (16) is satisfied, the fitness difference among the leading wolves is relatively large, so the weighted update in Equation (17) is adopted to enhance exploitation. Otherwise, the equal-weight update is used to maintain stable convergence.

(15) q(t)=g⋅a(t)

When

(16) $$f_{\delta }-f_{\alpha }>q(t)$$

it is indicated that the current population is still in a clearly stratified state, and a weighted update strategy based on fitness is adopted:

(17) $$X_{t+1}=\omega_{\alpha }X_{1}+\omega_{\beta }X_{2}+\omega_{\delta }X_{3}$$

where

(18) $$\omega_{i}=\frac{f_{\max}-f_{i}+\varphi }{\sum_{j\in \{\alpha ,\beta ,\delta \}}\left(f_{\max}-f_{i}+\phi \right)},\;i\in \{\alpha ,\beta ,\delta \}$$

$f_{\alpha }$, $f_{\beta }$, $f_{\delta }$ denote the fitness values of the alpha, beta, and delta wolves, respectively. $\omega_{i}$ denotes the normalized weight of the candidate position corresponding to the leader wolf *i*, $f_{i}$ is the fitness value of the corresponding leader wolf, and $f_{\max}$ denotes the maximum fitness value among the alpha, beta, and delta wolves. φ is a small positive constant used to avoid division by zero.

Otherwise, when the fitness gap within the population narrows, it indicates that the population is converging. In this case, equal-weighted average update should be adopted:

(19) $$X_{t+1}=\frac{X_{1}+X_{2}+X_{3}}{3}$$

where $X_{1}$, $X_{2}$, and $X_{3}$ denote the three candidate positions generated under the guidance of the alpha, beta, and delta wolves, respectively.

The core idea of this strategy is to strengthen the process of survival of the fittest when there are significant differences among the group members, and to achieve stable and average convergence when the group becomes homogeneous.

### 3.4. The QLFDGWO Algorithm

The required parameters for QLFDGWO include the problem dimension dim, population size pop, maximum number of iterations *T*_max_, and the fitness function *f*(*x*). The output result is the position value $X_{\alpha }$ of the leader wolf. The pseudocode of QLFDGWO is as follows.

The detailed procedure of the QLFDGWO algorithm is summarized in the pseudocode. First, the grey wolf population is initialized by Tent chaotic mapping, and the fitness values of all individuals are evaluated. The alpha wolf $X_{\alpha }$ is selected as the individual with the smallest fitness value, while $X_{\beta }$ and $X_{\delta }$ are selected using the fitness–distance-weighted leader selection mechanism. Then, the Q-table Q and the leader-weight Q-table $Q_{\omega }$ are initialized, and the candidate weight set Ω is defined. These operations correspond to Lines 1–7 in Algorithm 1.
**Algorithm 1. QLFDGWO****Input:** Dim, pop, ${\;T}_{\max}$, f(x)
**Output:** $X_{\alpha }$1: Initialize grey wolf population P using Tent chaotic mapping2: Calculate grey wolf positions fitness: fitness = f(x)
3: Select $X_{\alpha }$ as the wolf with the smallest fitness4: Select $X_{\beta }$ and $X_{\delta }$ using the fitness–distance-weighted leader selection mechanism5: Initialize Q-table: Q(s,a) = 0
6: Initialize leader-weight Q-table: $Q_{\omega }$(s, $a_{\omega }$) = 07: Define the weight candidate set Ω = {0.70, 0.80, 0.90, 0.98}8: For *t* < *T*_max_ do9:   Update a using Equation (4), update λ using Equation (6)10: Determine the current search state *s*11: Select ω from Ω using $Q_{\omega }$ and ε-greedy strategy12: Select $X_{\beta }$ and $X_{\delta }$ using Equations (10)–(13)13: For i < pop do14:  Determine state *s* using Equation (7)15:  Select action via ε-greedy using Equation (8)16:  **If** action = exploitation **then**17:   For *j* < dim do18:     Compute $X_{1},{\;X}_{2},\;X_{3}$ using Equation (2)19:   End for20:   Compute q using Equation (15)21:   If $|f_{\delta }-f_{\alpha }|>q$ then22:     Update *X_t_*_+1_ using Equation (17)23:   Else update *X_t_*_+1_ using Equation (19)24:   End if25:  Else update *X_t_*_+1_ using Equation (14)26:  End if27:  Evaluate new fitness, compute reward *R* using Equation (9)28:  Update Q-value using Equation (5)29:  Accept the new solution if improved 30: End for31: Update $X_{\alpha }$ according to the smallest fitness32: Update $X_{\beta }$ and $X_{\delta }$ using the FW leader selection mechanism33: Update $Q_{\omega }$ using fitness and diversity feedback34: End for35: Return $X_{\alpha }$


During the iterative optimization process, the convergence factor a and the dynamic threshold λ are updated, and the current search state is determined according to the predefined state rule. The weight coefficient ω is selected from Ω using the leader-weight Q-table $Q_{\omega }$ and the ε-greedy strategy. Based on the selected ω, $X_{\beta }$ and $X_{\delta }$ are further determined by the FW mechanism. These operations correspond to Lines 8–12 in Algorithm 1.

After the leader wolves are determined, each grey wolf is updated sequentially. For each individual, the search state is determined according to Equation (7), and the corresponding action is selected using the ε-greedy strategy. This allows the algorithm to adaptively switch between exploitation and exploration during the search process, as shown in Lines 13–15 in Algorithm 1.

When the exploitation action is selected, the candidate positions $X_{1},X_{2}$, and $X_{3}$ are calculated according to the GWO updating mechanism, and the dynamic threshold q is further calculated. According to the fitness difference between $X_{\alpha }$ and $X_{\delta }$, the algorithm chooses different exploitation update strategies to update $X_{t+1}$, as described in Lines 16–24. Otherwise, the exploration update strategy is adopted to improve population diversity and avoid premature convergence, corresponding to Lines 25–26 in Algorithm 1.

After each position is updated, the new fitness value is evaluated and the reward R is calculated. The Q-value is then updated using the Q-learning rule, and the improved solution is accepted if it outperforms the previous one. This process corresponds to Lines 27–29 in Algorithm 1. After all individuals are updated, $X_{\alpha }$ is updated according to the smallest fitness value, while $X_{\beta }$ and $X_{\delta }$ are reselected using the FW elite-guidance strategy. Meanwhile, $Q_{\omega }$ is updated using fitness and diversity feedback, as shown in Lines 31–33. When the termination condition is satisfied, the optimal solution $X_{\alpha }$ is returned, corresponding to Line 35 in Algorithm 1.

## 4. Experimental and Performance Analysis

### 4.1. Experimental Setup

To assess the global optimization performance of the QLFDGWO algorithm, CEC2017 benchmark functions were adopted for experimental testing. The experiments were conducted on 12 selected functions from the official CEC2017 benchmark suite, including unimodal, simple multimodal, hybrid, and composition functions. The selected functions are F1, F3, F4, F11, F12, F14, F15, F18, F20, F22, F25, and F28. Table 1 shows the type, name, domain and optimal solution of each test function. Additionally, in order to comprehensively evaluate the performance of the proposed QLFDGWO algorithm, five algorithms, namely PSO [5], GWO [17], MP-GWO [29], PSO-GWO [30], and WOA [31], were selected for comparative experiments.

During the testing phase, the overall size of the six algorithms is uniformly set to 30, and the maximum number of iterations is 500. The problem dimensions are set to 30 and 100 to test the performance of each algorithm. To ensure reproducibility and fairness, all algorithms were tested under the same search range, problem dimension, and termination criterion. All results were obtained from 30 independent runs. The experiments were implemented in MATLAB R2022b on a computer with an i7-1165G7 CPU and 8 GB RAM.

### 4.2. Experimental Results

**Figure 2:**
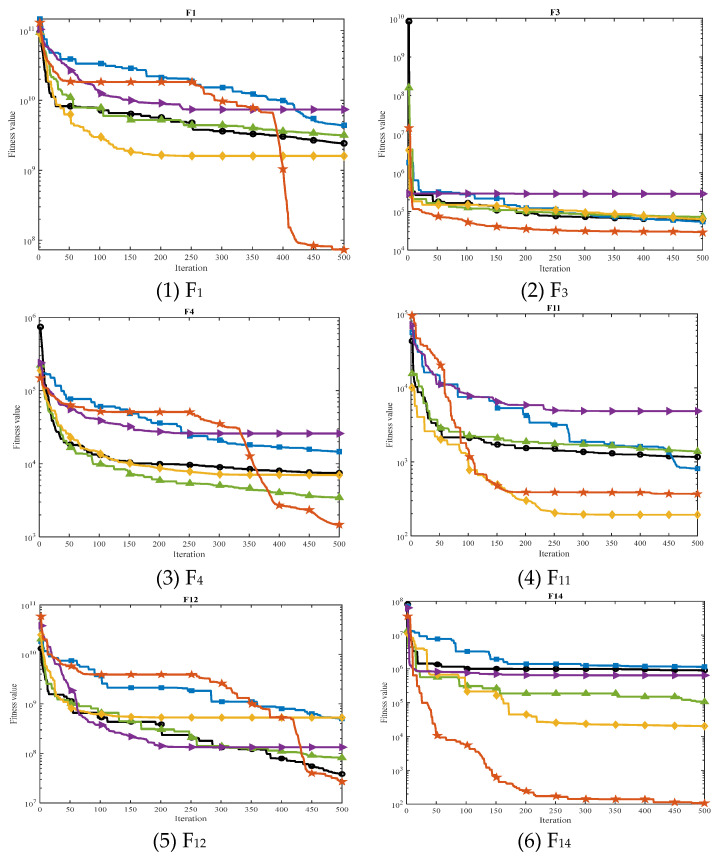

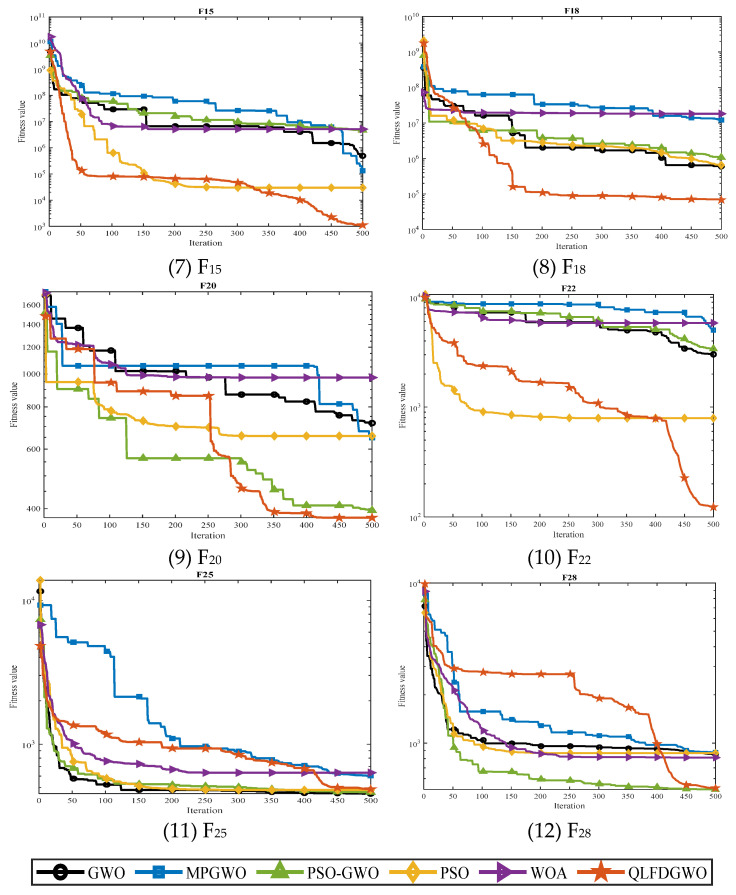
Convergence curves of six algorithms for the test functions (D = 30).

**Figure 3:**
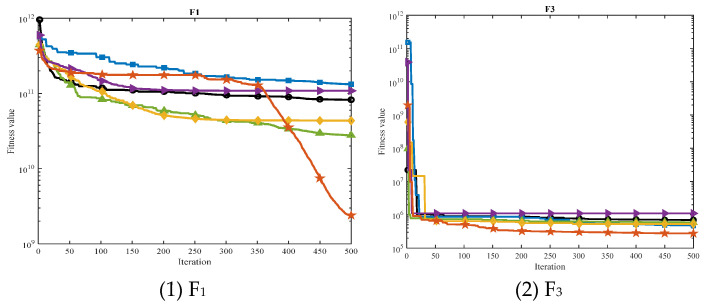

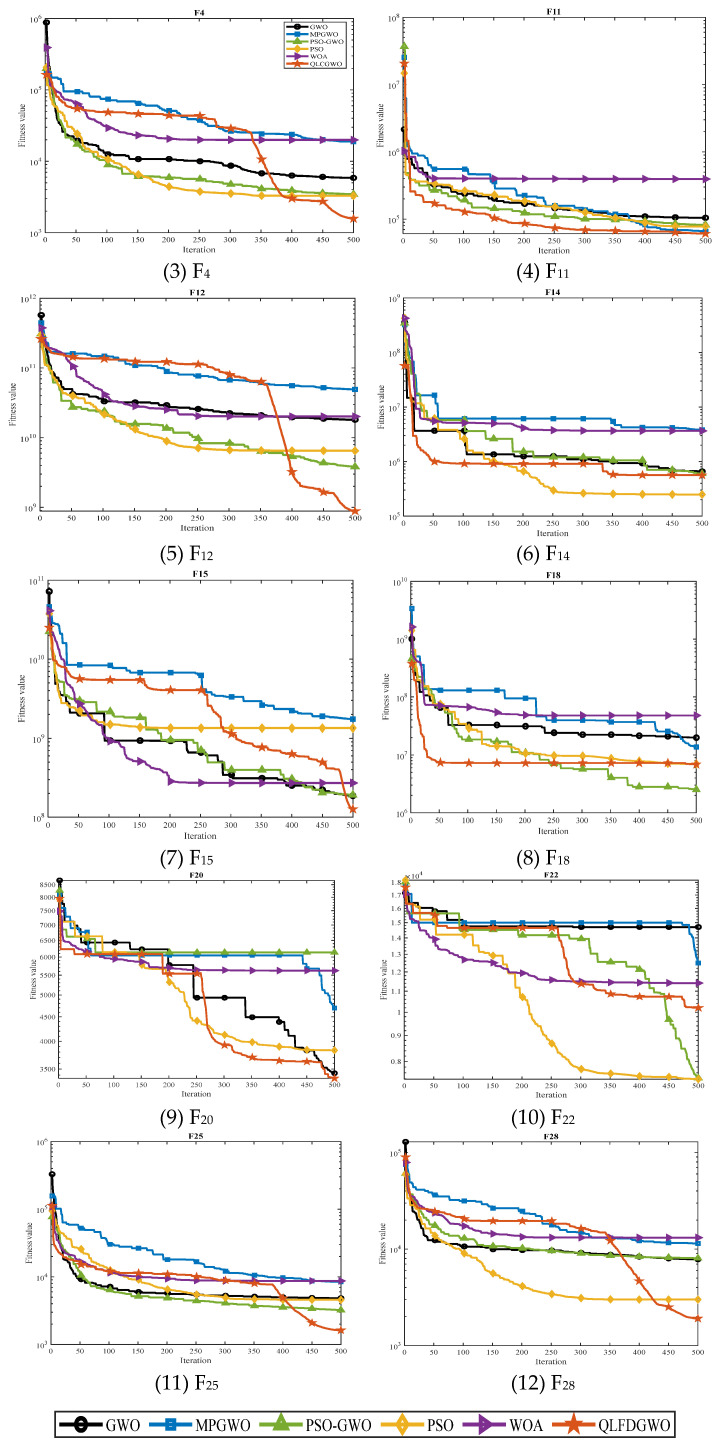
Convergence curves of six algorithms for the test functions (D = 100).

To analyze the search performance and convergence characteristics of different optimization algorithms during the solution process, the 12 selected CEC2017 benchmark functions were used to conduct comparative experiments on six algorithms. The experimental results are shown in Table 2 and Table 3. The convergence curves under D = 30 and D = 100 are shown in Figure 2 and Figure 3, respectively. The experiments were conducted at dimensions *D* = 30 and *D* = 100. By observing the fitness changes in each algorithm during the iterative process, the convergence speed, optimization accuracy, and global search ability of the algorithms can be intuitively reflected.

From the experimental results in Table 2 and Table 3, QLFDGWO achieves better or competitive optimization performance on most of the 12 selected CEC2017 benchmark functions. Under (D = 30), QLFDGWO significantly outperforms GWO on 11 functions, MP-GWO on 12 functions, PSO-GWO on 11 functions, PSO on nine functions, and WOA on 12 functions. These results indicate that the proposed algorithm has strong optimization accuracy in low-dimensional cases. In particular, QLFDGWO obtains smaller mean fitness values on most unimodal, multimodal, hybrid, and composition functions, showing its ability to balance global exploration and local exploitation.

For D = 100, QLFDGWO still maintains competitive performance as the problem dimension increases. It significantly outperforms GWO, MP-GWO, PSO-GWO, PSO, and WOA on eight, 10, eight, nine, and 11 functions, respectively. Although some compared algorithms obtain better or similar results on a few functions, such as F14, F20, F22, and F25, QLFDGWO achieves better overall performance on most high-dimensional test functions. This suggests that the proposed Q-learning-based strategy selection and fitness–distance-weighted leader selection mechanism can improve the robustness and scalability of GWO.

In terms of stability, QLFDGWO generally obtains smaller standard deviations on many functions, especially on F1, F3, F11, F12, and F18. This indicates that the proposed algorithm can maintain relatively consistent performance over 30 independent runs. Overall, the numerical results demonstrate that QLFDGWO has better optimization accuracy, stability, and robustness than the compared algorithms on most benchmark functions.

**Figure 4:**
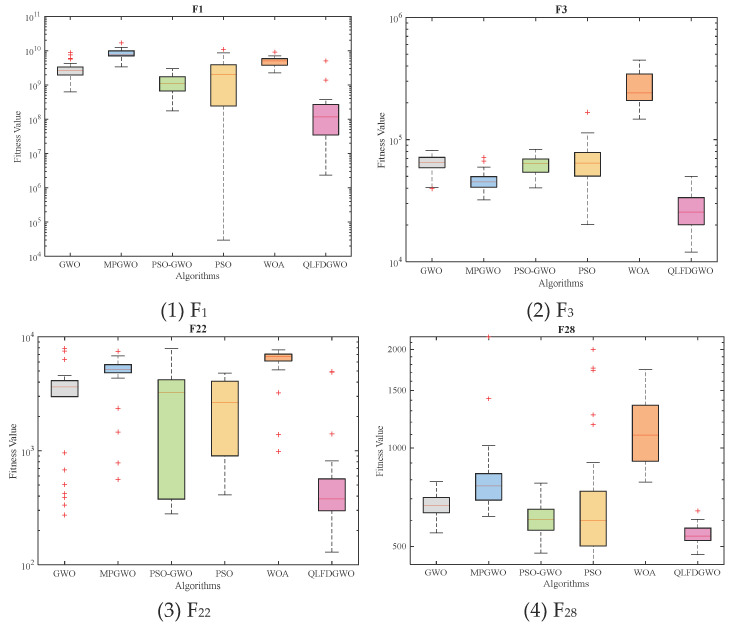
Box plot comparison of six algorithms (D = 30). The plus signs (+) indicate outliers obtained from independent runs.

Figure 4 presents the box plot comparison of the six algorithms on four representative benchmark functions under (D = 30). It can be observed that QLFDGWO generally obtains lower median fitness values than the compared algorithms, especially on F1, F3, and F22, indicating better optimization accuracy. In addition, the distribution range of QLFDGWO is relatively compact on most functions, which suggests that the proposed algorithm has good stability over multiple independent runs. Although some compared algorithms, such as PSO and PSO-GWO, may obtain competitive results on certain functions, their box ranges and whiskers are usually wider, showing larger fluctuations and weaker robustness. Overall, the box plot results further demonstrate that QLFDGWO can maintain better solution quality and more stable performance on different types of benchmark functions.

### 4.3. Ablation Experiment

#### 4.3.1. Ablation Experiment Design

To evaluate the contribution of different components in QLFDGWO, four algorithm variants were compared: GWO, GWO-Tent-a, QLFDGWO-noFD, and the complete QLFDGWO. GWO is the original baseline algorithm. GWO-Tent-a introduces Tent chaotic initialization and the cosine nonlinear convergence factor. QLFDGWO-noFD removes the fitness–distance-weighted leader selection mechanism, while QLFDGWO includes all proposed strategies. The experiments were conducted on F1, F12, and F18 under (D = 30) and (D = 100), as shown in Table 4 and Table 5. By comparing and analyzing the optimization results of different algorithm variants on these test functions, the contribution of each improvement strategy to the overall performance improvement of the QLFDGWO algorithm can be effectively evaluated.

#### 4.3.2. Experimental Results and Analysis

The results show that the optimization performance improves gradually as the proposed strategies are introduced. Compared with GWO, GWO-Tent-a obtains smaller mean values on most test functions, indicating that Tent chaotic initialization and the cosine convergence factor can improve population diversity and the exploration–exploitation transition. Furthermore, QLFDGWO-noFD performs better than GWO-Tent-a, showing that the Q-learning-based adaptive strategy contributes to better search performance. Finally, the complete QLFDGWO achieves the best mean values and smaller standard deviations on F1, F12, and F18 under both (D = 30) and (D = 100). This demonstrates that the fitness–distance-weighted leader selection mechanism further improves the guidance diversity of leader wolves and enhances the convergence accuracy and stability of the algorithm.

Overall, the ablation results confirm that each component contributes positively to the performance improvement of QLFDGWO, and the complete algorithm achieves the best overall optimization performance.

### 4.4. Computational Complexity Analysis

The computational complexity of QLFDGWO is analyzed in this section. Let N be the population size, D be the problem dimension, $T_{max}$ be the maximum number of iterations, and $C_{f}$ be the cost of one fitness evaluation.

In the initialization stage, Tent chaotic initialization generates N individuals with D dimensions, requiring (O(ND)) time. The initial fitness evaluation requires (O($NC_{f}$)). In each iteration, the cosine convergence factor, dynamic learning rate, and Q-learning update introduce only limited additional cost. Since the state and action spaces of Q-learning are fixed and small, the action selection and Q-table update cost is (O(N)) for the whole population.

The main computational cost comes from position updating, leader selection, and fitness evaluation. The exploration and exploitation position updates require (O(ND)). The fitness–distance-weighted leader selection requires distance calculation and candidate sorting, with complexity O(ND + NlogN). The fitness evaluation of the updated population requires (O($NC_{f}$)). Therefore, the total time complexity of QLFDGWO is [O(ND + $NC_{f}$ + $T_{max}$(ND + $NC_{f}$ + NlogN))].

For benchmark functions where the fitness evaluation cost is approximately proportional to the dimension, namely ($C_{f}$ = O(D)), the complexity can be simplified as [O($T_{max}$ND + $T_{max}$NlogN)].

Compared with the standard GWO, QLFDGWO mainly adds Q-learning-based strategy selection and fitness–distance-weighted leader selection. However, the Q-learning module uses a small, fixed Q-table and does not change the main complexity order. Thus, the dominant cost of QLFDGWO still comes from population updating and fitness evaluation. The space complexity is mainly determined by storing the population positions, so it is (O(ND)).

Therefore, the proposed mechanisms improve the adaptive search ability with acceptable computational overhead, making QLFDGWO applicable to high-dimensional benchmark optimization and UAV path planning scenarios.

### 4.5. Parameter Sensitivity Analysis

To verify the rationality of the Q-learning parameter settings, sensitivity analysis was conducted under (*D* = 30) on the 12 benchmark functions. Two key parameters were tested: the discount factor (γ) and the exploration probability (ε).

The results are shown in Table 6 and Table 7. Here, $f_{best}$ denotes the number of functions on which a parameter setting obtains the best result, and $r_{g}$ denotes the average rank. The *p*-value was calculated by the Wilcoxon signed-rank test between each parameter setting and the adopted default setting. A *p*-value smaller than 0.05 indicates a statistically significant difference. The results show that γ = 0.8 and ε = 0.1 achieve better overall rankings, indicating that the selected parameters can provide a suitable balance between exploration and exploitation. Therefore, these values are adopted in the following experiments.

The results show that QLFDGWO maintains relatively stable performance under different parameter settings. Among them, γ = 0.8 achieves a good balance between immediate and future rewards, while ε = 0.1 provides sufficient random exploration without weakening the learned strategy.

Overall, the selected parameter settings provide stable optimization performance across different types of functions, indicating that the performance improvement of QLFDGWO is not caused by a highly sensitive parameter configuration.

## 5. Case Study on UAV Path Planning

The planning area is described using a three-dimensional coordinate system. Let any point on the flight path in the three-dimensional space be represented as follows:

(20) P=(x,y,z)

where *x* and *y* represent the position coordinates of UAV on the horizontal plane, and *z* represents the flight altitude. The flight path is composed of multiple points on the flight path, namely

(21) $$Track=\left\{P_{1},P_{2},\cdots ,P_{n}\right\}$$

where $P_{i}=(x_{i},y_{i},z_{i})$ represents the *i*-th waypoint. The terrain is represented by an elevation matrix, and obstacles are modelled based on scene characteristics as mountains, cylindrical no-fly zones, or urban buildings.

### 5.1. Path Evaluation Function

The flight path of a UAV not only needs to ensure a safe clearance from the terrain but also needs to avoid cylindrical no-fly zones. Therefore, the comprehensive objective function for path planning is constructed as follows:

(22) $$F=w_{1}f_{len}+w_{2}f_{ter}+w_{3}f_{thr1}+w_{4}f_{thr2}+w_{5}f_{smo}$$

where *F* is the comprehensive fitness function of the path. $f_{len}$, $f_{ter}$, $f_{thr1}$, $f_{thr2}$ and $f_{smo}$ denote the path length cost, height cost, collision cost, no-fly zone threat cost, and smoothness cost, respectively. $w_{1}$, $w_{2}$, $w_{3}$, $w_{4}$ and $w_{5}$ are the corresponding weight coefficients.

(1) Path length cost function

The length cost is formulated as a normalized measure of the actual trajectory length relative to the Euclidean distance between the start and end points:

(23) $$f_{len}=\frac{L}{L_{straight}}$$

where *L* represents the actual flight path length, while $L_{\mathrm{straight}}$ is the Euclidean straight-line distance between the starting point and the ending point.

(2) Path height cost function

Let the terrain elevation be $h\left(x,y\right)$, then the relative terrain clearance height $h_{c}(i)$ of the tracking waypoint Pi is defined as

(24) $$h_{c}(i)=z_{i}-h\left(x_{i},y_{i}\right)$$

Path height cost function is given by

(25) $$f_{ter}=\frac{1}{n}\sum_{i=1}^{n}\phi_{ter}(i)$$

where

(26) $$\phi_{ter}(i)=\left\{\begin{array}{l} {\left(\frac{h_{\min}-h_{c}(i)}{h_{\min}}\right)}^{2},0\leq h_{c}(i)<h_{\min}\\ 0,h_{c}(i)\geq h_{\min} \end{array}\right.$$

where $h_{\min}$ is the minimum height. When $h_{c}(i)<0$, it indicates that the track has collided with the terrain.

(3) Collision Cost Function

The cost of collision can be expressed as

(27) $$f_{\mathrm{thr}1}=\frac{1}{N_{p}}\left(\sum_{i=1}^{N_{p}}\phi (i)\right)$$

where $N_{p}$ represents the number of path control points. ϕ(i) represents the collision penalties of control point *i*.

(28) $$\phi_{\left(i\right)}=\left\{\begin{array}{l} 3{\left(\frac{d_{soft}-d_{b}(i)}{d_{soft}}\right)}^{2},0\leq d_{b}(i)<d_{soft}\\ 0,d_{b}(i)\geq d_{soft} \end{array}\right.$$

where $d_{b}(i)$ denotes the minimum distance from the *i*-th path control point to the obstacle boundary, and $d_{soft}$ is the soft safety distance threshold.

(4) No-fly zone threat cost function

The no-fly zone is described using a cylindrical model. Let the centre of the m-th no-fly zone in the horizontal plane be $c_{m}$, and its radius be $r_{m}$. For any flight path segment, calculate the minimum planar distance $d_{seg}(i,m)$ from it to the centre of the no-fly zone, and define the margin of safety of the relative safety boundary as follows:

(29) $$d_{i,m}=d_{seg}(i,m)-(r_{m}+d_{b})$$

where $d_{b}$ represents the additional buffer distance. Thus, the threat cost is defined as

(30) $$f_{thr2}=\frac{1}{N_{s}}\sum_{i=1}^{n-1}\sum_{m=1}^{M}\phi_{thr}(i,m)$$

where $N_{s}$ denotes the total number of flight path segments, and *M* represents the number of no-fly zones.

(31) $$\phi_{\mathrm{thr}}(i,m)=\left\{\begin{array}{l} {\left(\frac{d_{soft}-d_{i,m}}{d_{soft}}\right)}^{2},0\leq d_{i,m}<d_{soft}\\ 0,d_{i,m}\geq d_{soft} \end{array}\right.$$

where $d_{soft}$ denotes the soft safety distance threshold. If $d_{i,m}<0$, the flight segment has intruded into the restricted area.

(5) Path smooth cost function

To prevent sharp turns in the trajectory, a discrete second-order difference is used to measure the smoothness of the trajectory:

(32) $$f_{smo}=\frac{1}{n-2}\sum_{i=2}^{n-1}{\left\|P_{i+1}-2P_{i}+P_{i-1}\right\|}^{2}$$

where $P_{i}$ represents the *i*-th waypoint of the UAV path, and *n* is the total number of waypoints. The second-order difference ${\left\|P_{i+1}-2P_{i}+P_{i-1}\right\|}^{2}$ is used to evaluate the curvature variation in the path.

### 5.2. Simulation Results

To verify the effectiveness of the proposed QLFDGWO algorithm for the problem of unmanned aerial vehicle (UAV) path planning, a multi-algorithm comparison simulation experiment was conducted in six complex scenarios [32] including mountainous areas, urban areas, prohibited flight zones, etc. The comparison algorithms included PSO [5], GWO [17], MP-GWO [29], PSO-GWO [30], and WOA [31]. The experimental results included the optimal value, average value, standard deviation, average running time, and success rate.

**Figure 5:**
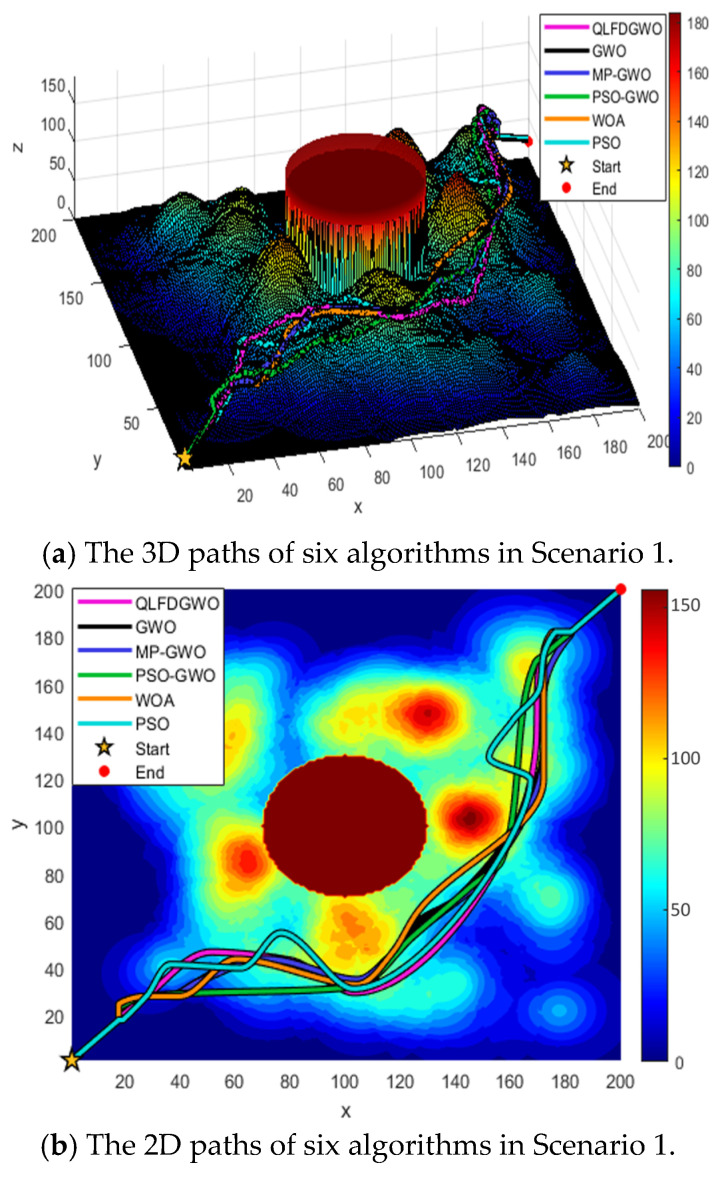
The paths of six algorithms in Scenario 1.

**Figure 6:**
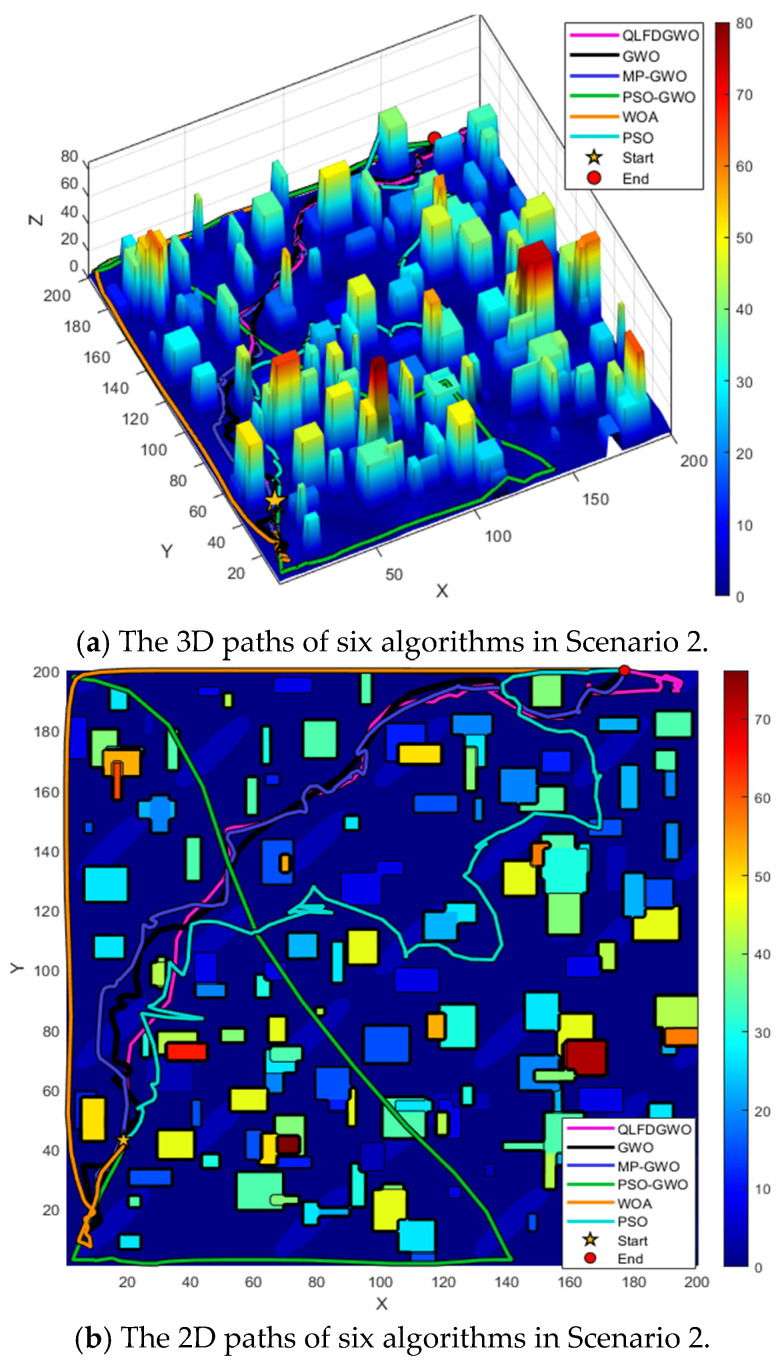
The paths of six algorithms in Scenario 2.

**Figure 7:**
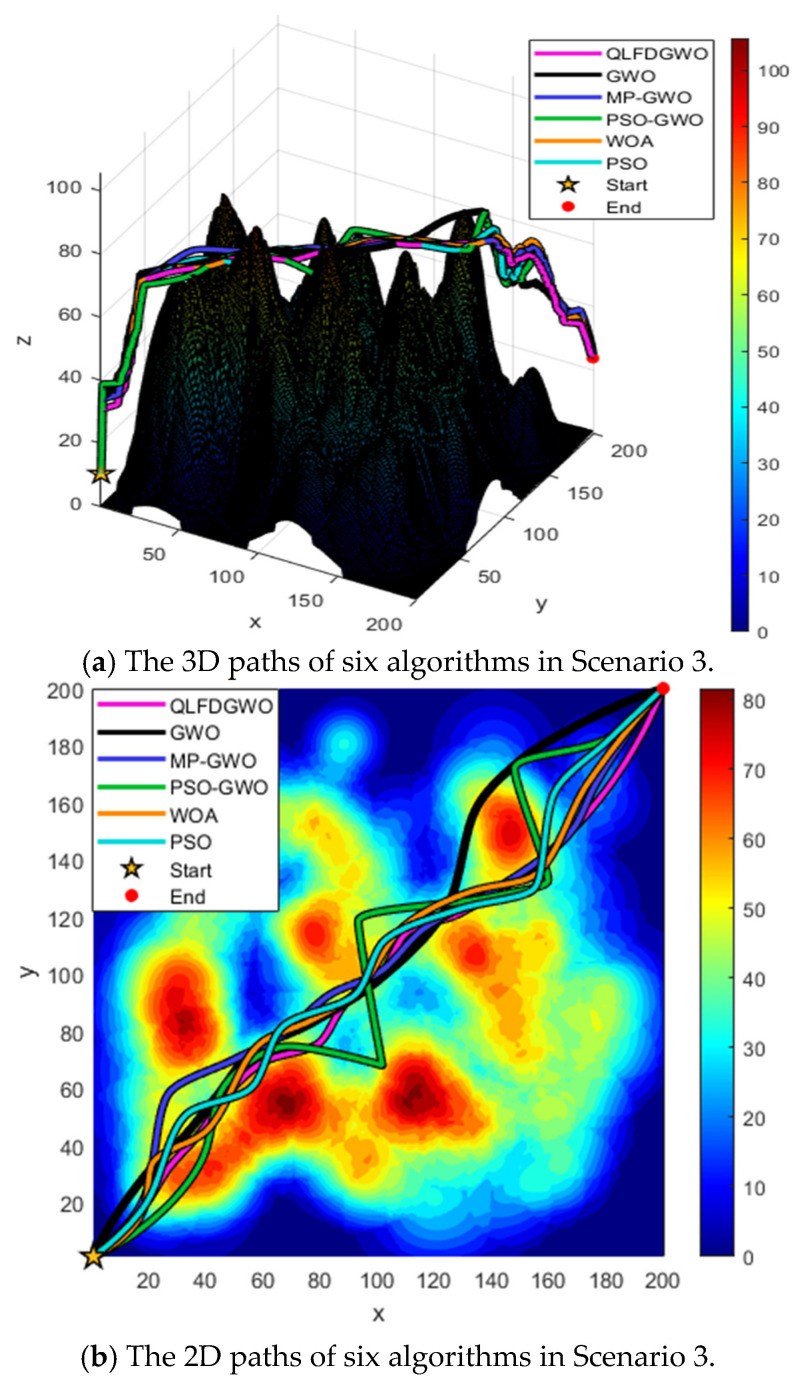
The paths of six algorithms in Scenario 3.

**Figure 8:**
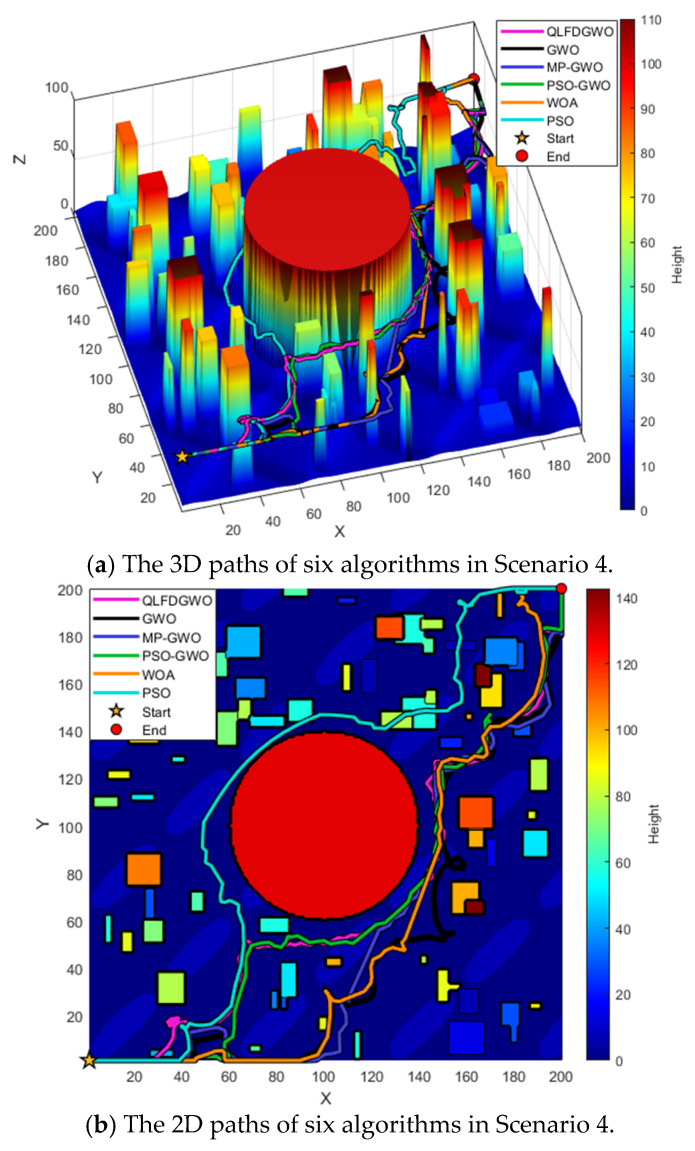
The paths of six algorithms in Scenario 4.

**Figure 9:**
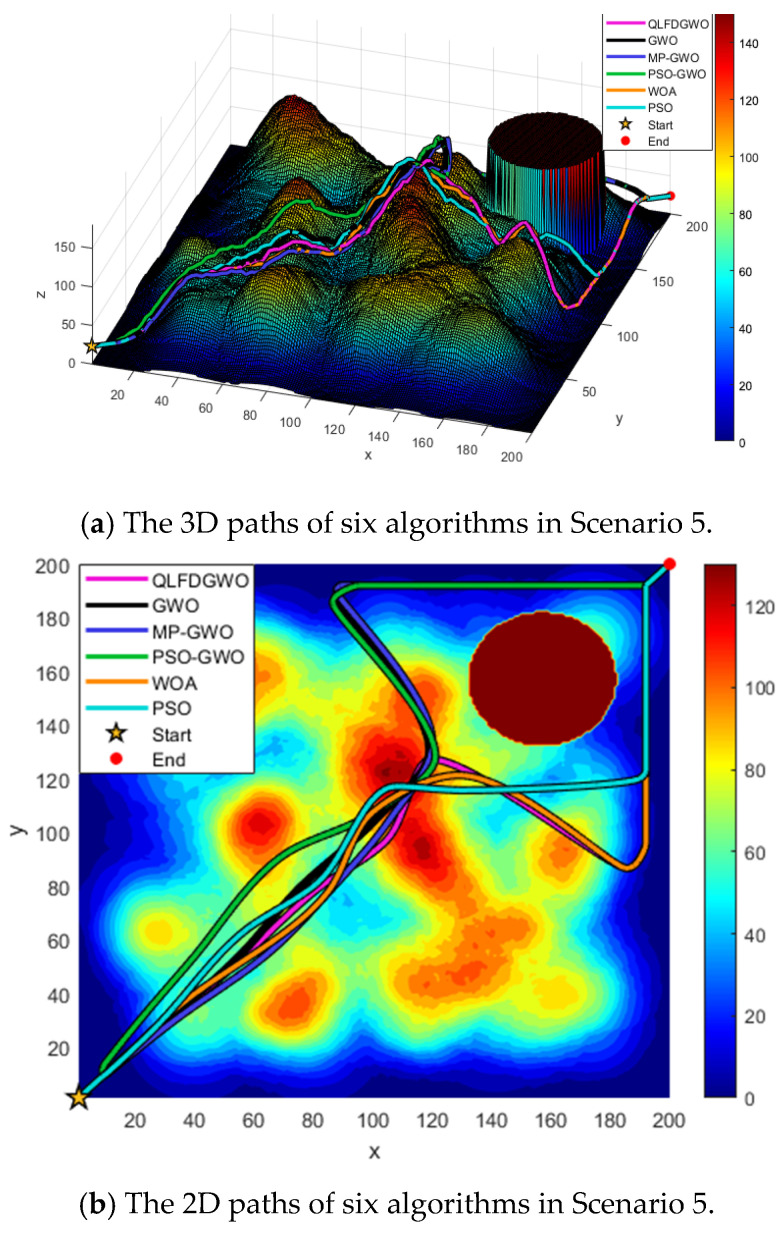
The paths of six algorithms in Scenario 5.

**Figure 10:**
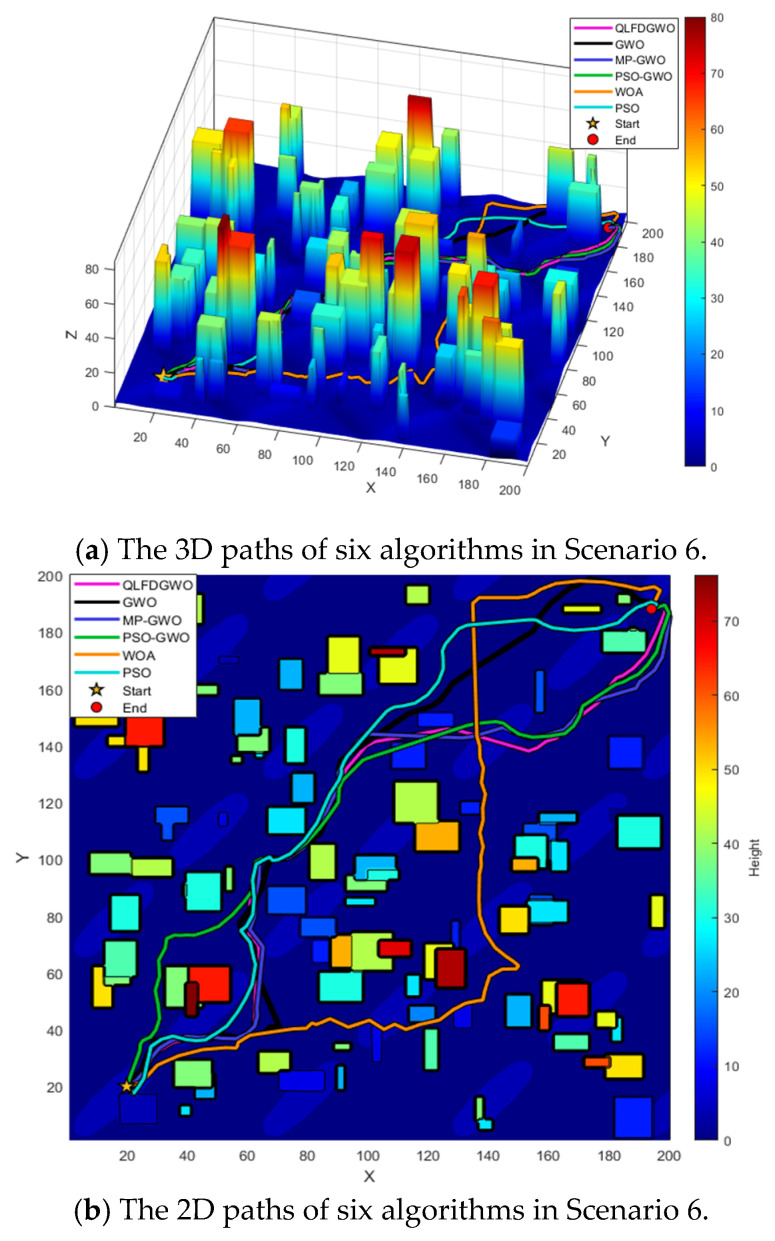
The paths of six algorithms in Scenario 6.

**Figure 11:**
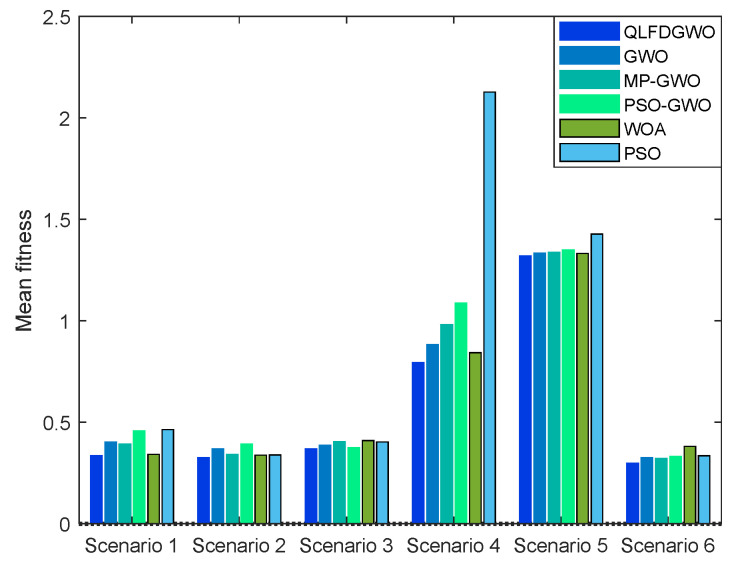
The mean fitness values of different algorithms in six different scenarios.

To provide consistent experimental conditions, each algorithm used the same starting and ending positions, environmental parameters, and constraints in the same scenario. The number of individuals in the population was uniformly set to 30, and the maximum number of iterations was set to 100. In each scenario, every algorithm was independently executed 30 times, and statistical analysis was performed on the results to minimize the effect of random fluctuations. The experimental results are shown in Table 8, which includes the best fitness value, mean fitness value, standard deviation, average running time and success rate. The 3D and 2D paths in six scenarios are shown in Figure 5, Figure 6, Figure 7, Figure 8, Figure 9 and Figure 10. Figure 11 presents the comparison results of the average fitness values.

From the experimental results of Table 8, it can be seen that the proposed QLFDGWO algorithm demonstrates overall better convergence performance, path planning quality, and environmental adaptability. Compared with the five contrast algorithms, except for the optimal fitness in Scenario 1, QLFDGWO can obtain lower optimal fitness and average fitness in most scenarios, with a smaller standard deviation, indicating that it not only has a strong optimization ability but also has more stable results after multiple independent runs.

In the mountain environment of Scenario 1, although WOA obtains a slightly smaller best value, QLFDGWO achieves a better mean value and smaller standard deviation, indicating better overall stability. PSO-GWO and PSO have larger average values and more obvious fluctuations, indicating that their solution quality and stability are relatively weak. Compared with the mountain scenario, in the urban scenario of Scenario 2, the distribution of buildings is denser and the passable space is narrower, so the constraints of the path planning problem are stronger and the search difficulty is higher. In 20 independent runs, the success rates of PSO-GWO and PSO are 60% and 40%, respectively, indicating weaker robustness in generating feasible paths under dense urban constraints. However, QLFDGWO can stably obtain collision-free feasible paths, demonstrating stronger robustness and environmental adaptability. In Scenario 3, the optimal value, average value, and standard deviation of QLFDGWO are all at the top; PSO-GWO is second, but its variance is larger, indicating that the algorithm is not stable. The WOA obtains larger mean values, indicating that its optimization ability and stability are not good enough. In Scenario 4, the average and standard deviation of PSO-GWO and PSO are relatively high, indicating that the solution’s volatility is greater and the stability is relatively insufficient. QLFDGWO can search for feasible paths that meet safety constraints in Scenarios 5 and 6.

**Figure 12:**
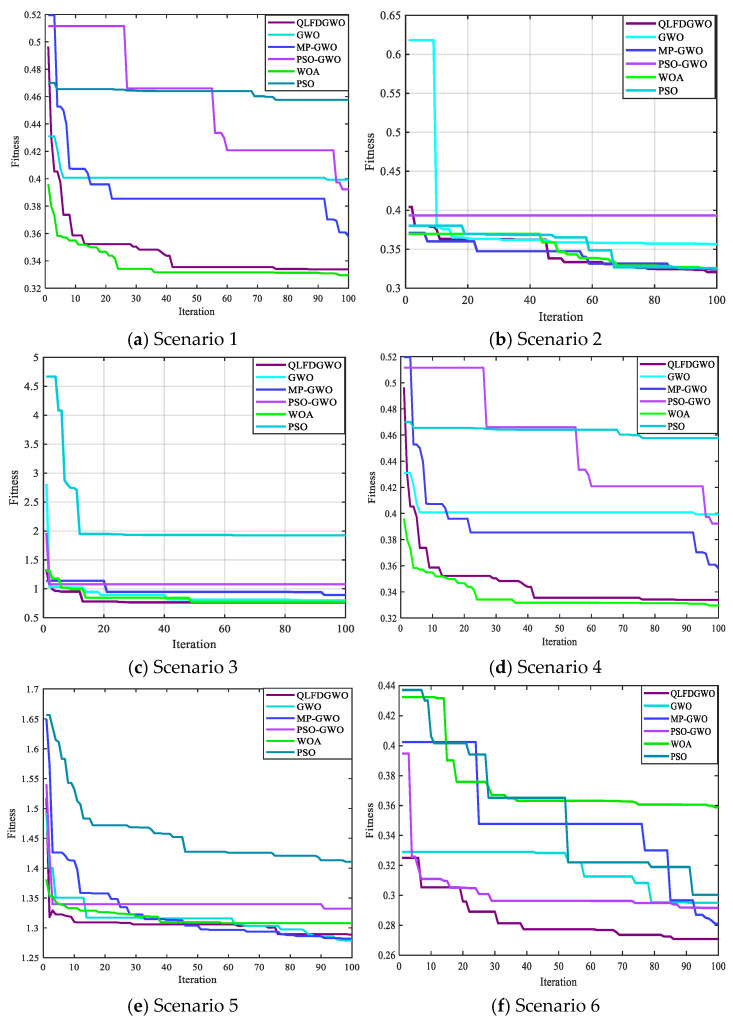
Convergence curves of different algorithms in 6 scenarios.

Figure 5, Figure 6, Figure 7, Figure 8, Figure 9 and Figure 10 display that QLFDGWO can generate safe and feasible flight paths in different scenarios. Its planning trajectory is smoother overall, with less detour distance and better directionality and continuity, and can maintain better path quality while avoiding constraints such as terrain, buildings, and no-fly zones. In contrast, some comparison algorithms have problems such as excessive detour, trajectory jitter, and approaching the boundary of obstacles, indicating that their search ability for feasible solutions under complex constraints and robustness are relatively insufficient. From the convergence curve diagrams in Figure 12, it can be seen that QLFDGWO can usually quickly reduce the target function value in the early iterations and maintain a good convergence trend in subsequent iterations, demonstrating strong global search and local development balance ability.

Although the improvement strategies will incur certain computational costs, the average running time of QLFDGWO in various scenarios remains within an acceptable range and does not significantly increase the computational burden. Overall, QLFDGWO demonstrates superior comprehensive advantages in terms of path quality, convergence speed, stability, safety, and robustness. This verifies the effectiveness of the proposed improvement strategies in solving complex 3D UAV path planning problems.

## 6. Conclusions

This paper proposes an improved grey wolf optimization algorithm QLFDGWO that integrates the Q-learning mechanism. The Q-learning-based strategy selection mechanism enables adaptive switching between exploration and exploitation, while the fitness–distance-weighted leader selection strategy improves the diversity of leader guidance by selecting beta and delta wolves according to both fitness quality and spatial distribution. In addition, the dynamic threshold-weighted update strategy enhances the targeting of population position updates, thereby improving convergence accuracy and stability. Tent chaotic initialization and the cosine nonlinear convergence factor further improve the initial population distribution and the stage transition of the iterative search process. To verify the effectiveness of the proposed algorithm, QLFDGWO was systematically compared with the classic GWO, PSO-GWO, and MP-GWO, as well as the two classic swarm intelligence algorithms PSO and WOA, and simulation experiments were conducted on 12 typical functions in the CEC2017 test function set. The experimental results show that QLFDGWO exhibits superior convergence performance and stronger stability in single-peak, multi-peak, low-dimensional, and high-dimensional optimization problems, verifying the effectiveness of the proposed improvement strategies in enhancing the global search ability and local development ability. QLFDGWO was applied to the unmanned aerial vehicle trajectory planning problem and path planning experiments were carried out in different simulation environments. The results show that QLFDGWO can obtain higher-quality feasible paths under complex terrain and obstacle constraints, and the generated trajectories have lower objective function values, greater safety margins, and shorter flight distances and flight times, demonstrating good engineering applicability and practical value.

Although the proposed QLFDGWO algorithm achieves good performance in benchmark testing and UAV path planning, it still has some limitations. Due to the introduction of Q-learning-based strategy selection, fitness–distance-weighted leader selection, and dynamic threshold-weighted update mechanisms, the algorithm structure is more complex than that of the original GWO, which may introduce additional computational overhead in some scenarios. Moreover, the current UAV path planning experiments are mainly conducted in simulated environments, and the adaptability of the proposed method to dynamic obstacles, uncertain disturbances, and real-world flight scenarios still requires further verification. Future work will focus on reducing computational complexity, improving real-time performance, and extending the proposed method to more complex dynamic path planning tasks.

## Figures and Tables

**Table 1:** Test functions.

Type	No	Function Name	Range	Opt.
Unimodal Functions	F1	Shifted and Rotated Bent Cigar Function	[−100, 100]	100
Simple Multimodal Functions	F3	Shifted and Rotated Rosenbrock Function	[−100, 100]	300
	F4	Shifted and Rotated Rastrigin Function	[−100, 100]	400
Hybrid Functions	F11	Hybrid Functions 2 (N = 3)	[−100, 100]	1100
	F12	Hybrid Functions 3 (N = 3)	[−100, 100]	1200
	F14	Hybrid Functions 5 (N = 4)	[−100, 100]	1400
	F15	Hybrid Functions 6 (N = 4)	[−100, 100]	1500
	F18	Hybrid Functions 6 (N = 5)	[−100, 100]	1800
Composition Functions	F20	Composition Function 1 (N = 3)	[−100, 100]	2000
	F22	Composition Function 3 (N = 4)	[−100, 100]	2200
	F25	Composition Function 6 (N = 5)	[−100, 100]	2500
	F28	Composition Function 9 (N = 3)	[−100, 100]	2800

**Table 2:** Results of six algorithms (D = 30).

Fun.	QLFDGWO	GWO	MP-GWO	PSO-GWO	PSO	WOA
	Mean Std	Mean Std	Mean Std	Mean Std	Mean Std	Mean Std
F1	**3.690 × 10^8^** **9.848 × 10^3^**	2.849 × 10^9^ 1.614 × 10^9^ (+)	8.237 × 10^9^ 4.381 × 10^9^ (+)	1.320 × 10^9^ 1.044 × 10^9^ (+)	2.768 × 10^9^ 3.707 × 10^9^ (+)	5.120 × 10^9^ 1.785 × 10^9^ (+)
F3	**2.886 × 10^5^** **3.848 × 10^4^**	6.593 × 10^5^ 7.524 × 10^4^ (+)	5.582 × 10^5^ 6.253 × 10^4^ (+)	6.495 × 10^5^ 8.156 × 10^4^ (+)	7.149 × 10^5^ 1.067 × 10^5^ (+)	1.164 × 10^6^ 1.668 × 10^5^ (+)
F4	**1.707 × 10^2^** **2.037 × 10^1^**	1.763 × 10^2^ 1.402 × 10^2^ (=)	4.109 × 10^2^ 2.463 × 10^2^ (+)	2.430 × 10^2^ 1.775 × 10^2^ (+)	2.891 × 10^2^ 1.224 × 10^2^ (+)	1.011 × 10^3^ 3.493 × 10^2^ (+)
F11	6.250 × 10^2^ 2.158 × 10^1^	2.487 × 10^3^ 7.251 × 10^1^ (+)	8.703 × 10^2^ 9.923 × 10^1^ (+)	2.915 × 10^3^ 4.831 × 10^1^ (+)	**3.040 × 10^2^** **7.280 × 10^1^ (−)**	9.974 × 10^3^ 1.223 × 10^2^ (+)
F12	**3.404 × 10^7^** **1.302 × 10^6^**	5.584 × 10^8^ 5.668 × 10^7^ (+)	2.520 × 10^8^ 1.164 × 10^8^ (+)	4.683 × 10^7^ 4.394 × 10^7^ (+)	4.559 × 10^8^ 1.945 × 10^7^ (+)	8.383 × 10^8^ 2.481 × 10^8^ (+)
F14	**1.632 × 10^2^** **1.493 × 10^2^**	1.053 × 10^5^ 1.368 × 10^6^ (+)	1.534 × 10^5^ 2.177 × 10^7^ (+)	2.052 × 10^5^ 4.540 × 10^5^ (+)	1.019 × 10^5^ 8.167 × 10^4^ (+)	5.615 × 10^5^ 2.248 × 10^5^ (+)
F15	**2.861 × 10^4^** **1.891 × 10^4^**	4.278 × 10^4^ 1.517 × 10^6^ (+)	6.174 × 10^7^ 1.641 × 10^4^ (+)	5.435 × 10^4^ 4.385 × 10^5^ (+)	6.054 × 10^4^ 1.934 × 10^4^ (+)	5.315 × 10^6^ 5.315 × 10^6^ (+)
F18	**4.105 × 10^2^** **2.069 × 10^1^**	1.269 × 10^6^ 2.543 × 10^4^ (+)	2.452 × 10^6^ 2.887 × 10^1^ (+)	5.259 × 10^6^ 2.117 × 10^1^ (+)	8.448 × 10^5^ 4.364 × 10^1^ (+)	8.59 × 10^5^ 7.910 × 10^1^ (+)
F20	**3.719 × 10^2^** **7.830 × 10^1^**	4.593 × 10^2^ 1.455 × 10^2^ (+)	4.353 × 10^2^ 1.778 × 10^2^ (+)	5.824 × 10^2^ 2.251 × 10^2^ (+)	7.195 × 10^2^ 2.467 × 10^2^ (+)	1.031 × 10^3^ 1.543 × 10^2^ (+)
F22	**1.006 × 10^3^** **1.663 × 10^3^**	2.829 × 10^3^ 2.547 × 10^3^ (+)	4.165 × 10^3^ 1.892 × 10^3^ (+)	2.527 × 10^3^ 2.040 × 10^3^ (+)	2.208 × 10^3^ 1.782 × 10^3^ (+)	5.515 × 10^3^ 2.095 × 10^3^ (+)
F25	4.798 × 10^2^ 4.620 × 10^1^	5.298 × 10^2^ 2.816 × 10^1^ (+)	6.663 × 10^2^ 5.987 × 10^1^ (+)	**4.434 × 10^2^** **6.285 × 10^1^ (−)**	4.633 × 10^2^ 2.719 × 10^1^ (=)	7.110 × 10^2^ 7.273 × 10^1^ (+)
F28	**5.504 × 10^2^** **6.342 × 10^1^**	7.185 × 10^2^ 1.444 × 10^2^ (+)	8.303 × 10^2^ 2.720 × 10^2^ (+)	6.056 × 10^2^ 8.647 × 10^1^ (+)	5.674 × 10^2^ 1.307 × 10^2^ (=)	1.235 × 10^3^ 3.664 × 10^2^ (+)
+(QLFDGWO is better)		11	12	11	9	12
−(QLFDGWO is worse)		0	0	1	1	0
=		1	0	0	2	0
rank	1.25	3.58	4.25	3.33	3.00	5.58

**Table 3:** Results of six algorithms (D = 100).

Fun.	QLFDGWO	GWO	MP-GWO	PSO-GWO	PSO	WOA
	Mean Std	Mean Std	Mean Std	Mean Std	Mean Std	Mean Std
F1	**1.652 × 10^9^** **3.857 × 10^8^**	5.534 × 10^10^ 6.145 × 10^9^ (+)	1.327 × 10^11^ 1.234 × 10^10^ (+)	2.813 × 10^10^ 6.508 × 10^9^ (+)	5.854 × 10^10^ 1.006 × 10^10^ (+)	1.082 × 10^11^ 1.070 × 10^10^ (+)
F3	**2.408 × 10^5^** **1.024 × 10^5^**	6.788 × 10^5^ 1.275 × 10^5^ (+)	5.132 × 10^5^ 1.146 × 10^5^ (+)	6.581 × 10^5^ 5.334 × 10^5^ (+)	4.143 × 10^5^ 1.336 × 10^5^ (+)	7.106 × 10^5^ 1.386 × 10^5^ (+)
F4	**1.572 × 10^3^** **1.692 × 10^2^**	5.846 × 10^3^ 1.255 × 10^3^ (+)	1.881 × 10^4^ 3.769 × 10^3^ (+)	3.455 × 10^3^ 8.279 × 10^2^ (+)	3.284 × 10^3^ 2.676 × 10^3^ (+)	1.989 × 10^4^ 3.999 × 10^3^ (+)
F11	**4.987 × 10^4^** **1.026 × 10^2^**	9.741 × 10^4^ 1.037 × 10^2^ (+)	6.637 × 10^4^ 1.641 × 10^2^ (+)	6.983 × 10^4^ 1.053 × 10^2^ (+)	5.588 × 10^4^ 3.009 × 10^2^ (+)	2.176 × 10^5^ 1.955 × 10^2^ (+)
F12	**1.504 × 10^9^** **1.061 × 10^8^**	1.992 × 10^10^ 2.444 × 10^9^ (+)	4.199 × 10^10^ 4.874 × 10^9^ (+)	9.500 × 10^9^ 5.069 × 10^9^ (+)	7.688 × 10^9^ 1.180 × 10^10^ (+)	2.876 × 10^10^ 7.006 × 10^9^ (+)
F14	2.410 × 10^6^ 1.216 × 10^6^	2.068 × 10^6^ 8.913 × 10^6^ (−)	6.264 × 10^6^ 6.876 × 10^6^ (+)	**4.350 × 10^5^** **3.807 × 10^5^ (−)**	3.336 × 10^6^ 1.502 × 10^6^ (+)	1.318 × 10^7^ 4.355 × 10^6^ (+)
F15	**1.109 × 10^8^** **7.268 × 10^7^**	5.247 × 10^8^ 5.286 × 10^8^ (+)	2.506 × 10^9^ 2.024 × 10^9^ (+)	7.961 × 10^8^ 4.292 × 10^8^ (+)	2.067 × 10^9^ 9.077 × 10^8^ (+)	3.389 × 10^8^ 2.048 × 10^8^ (+)
F18	**8.575 × 10^6^** **1.628 × 10^5^**	1.232 × 10^7^ 6.372 × 10^6^ (+)	1.959 × 10^7^ 1.027 × 10^6^ (+)	9.735 × 10^6^ 1.533 × 10^6^ (+)	1.855 × 10^6^ 1.644 × 10^6^ (+)	1.662 × 10^7^ 5.979 × 10^5^ (+)
F20	**3.090 × 10^3^** **2.088 × 10^2^**	3.335 × 10^3^ 1.114 × 10^3^ (=)	3.582 × 10^3^ 4.970 × 10^2^ (=)	5.595 × 10^3^ 2.402 × 10^2^ (+)	4.011 × 10^3^ 2.132 × 10^2^ (=)	6.109 × 10^3^ 2.805 × 10^2^ (+)
F22	2.673 × 10^4^ 3.328 × 10^3^	1.848 × 10^4^ 6.469 × 10^3^ (−)	2.851 × 10^4^ 1.227 × 10^4^ (=)	3.297 × 10^4^ 1.650 × 10^4^ (=)	**1.764 × 10^4^** **4.775 × 10^4^ (−)**	2.940 × 10^4^ 5.476 × 10^3^ (=)
F25	**1.764 × 10^3^** **2.489 × 10^2^**	3.602 × 10^3^ 8.466 × 10^2^ (=)	6.280 × 10^3^ 2.317 × 10^3^ (+)	3.127 × 10^3^ 5.405 × 10^2^ (=)	2.295 × 10^3^ 2.884 × 10^3^ (=)	7.735 × 10^3^ 3.356 × 10^2^ (+)
F28	**1.813 × 10^3^** **6.123 × 10^2^**	8.170 × 10^3^ 1.853 × 10^3^ (+)	1.212 × 10^4^ 2.224 × 10^3^ (+)	3.341 × 10^3^ 1.136 × 10^3^ (=)	7.283 × 10^3^ 2.489 × 10^3^ (+)	1.223 × 10^4^ 1.269 × 10^3^ (+)
+(QLFDGWO is better)		8	10	8	9	11
−(QLFDGWO is worse)		2	0	1	1	0
=		2	2	3	2	1
rank	1.42	3.50	4.75	3.33	2.67	5.33

**Table 4:** Comparison results of test at *D* = 30.

Func.	Metrics	Gwo	GWO-Tent-a	QLFDGWO-noFD	QLFDGWO
F1	Mean	2.849 × 10^9^	7.644 × 10^8^	6.039 × 10^8^	**3.690 × 10^8^**
	Std	1.614 × 10^9^	7.413 × 10^7^	8.721 × 10^4^	**9.848 × 10^3^**
F12	Mean	5.584 × 10^8^	2.774 × 10^8^	6.332 × 10^7^	**3.404 × 10^7^**
	Std	5.668 × 10^7^	1.941 × 10^7^	3.470 × 10^6^	**1.302 × 10^6^**
F18	Mean	1.269 × 10^6^	1.644 × 10^5^	2.330 × 10^4^	**4.105 × 10^2^**
	Std	2.543 × 10^4^	1.029 × 10^4^	2.636 × 10^3^	**2.069 × 10^1^**

**Table 5:** Comparison results of test at D = 100.

Func.	Metrics	GWO	GWO-Tent-a	QLFDGWO-noFD	QLFDGWO
F1	Mean	5.534 × 10^10^	5.027 × 10^10^	6.844 × 10^9^	**1.652 × 10^9^**
	Std	6.145 × 10^9^	1.639 × 10^9^	1.208 × 10^9^	**3.857 × 10^8^**
F12	Mean	1.992 × 10^10^	8.449 × 10^9^	7.028 × 10^9^	**1.504 × 10^9^**
	Std	2.444 × 10^9^	1.352 × 10^9^	8.710 × 10^8^	**1.061 × 10^8^**
F18	Mean	1.232 × 10^7^	9.305 × 10^6^	9.104 × 10^6^	**8.575 × 10^6^**
	Std	6.372 × 10^6^	4.917 × 10^6^	7.383 × 10^5^	**1.628 × 10^5^**

**Table 6:** Sensitivity analysis of γ.

γ	0.1	0.2	0.3	0.4	0.5	0.6	0.7	0.8	0.9
$f_{best}$	4	4	5	4	5	5	6	8	5
$r_{g}$	5.324	6.217	6.043	6.310	5.635	5.139	5.012	4.316	5.872
*p*-value	0.0295	0.0310	0.0336	0.0403	0.0894	0.1121	0.0539	-	0.3024
*p* < 0.05	YES	YES	YES	YES	NO	NO	NO	-	NO

**Table 7:** Sensitivity analysis of ε.

ε	0.1	0.2	0.3	0.4	0.5	0.6	0.7	0.8	0.9
$f_{best}$	7	5	6	5	4	4	2	1	1
$r_{g}$	5.108	5.396	5.224	5.797	6.128	6.325	6.764	6.934	6.941
*p*-value	-	0.0856	0.1429	0.0328	0.0917	0.0412	0.0199	0.0377	0.0411
*p* < 0.05	-	NO	NO	YES	NO	YES	YES	YES	YES

**Table 8:** The results of different algorithms in six scenarios.

Scenario	Algorithm	Best	Mean	Std	Mean Time (s)	Success Rate
1	QLFDGWO	0.332	**0.334**	**0.013**	172.57	96.67%
	GWO	0.399	0.402	0.019	169.09	93.33%
	MP-GWO	0.358	0.392	0.024	**157.96**	86.67%
	PSO-GWO	0.392	0.457	0.038	172.09	80.00%
	WOA	**0.330**	0.341	0.023	170.77	90.00%
	PSO	0.458	0.463	0.021	165.00	86.67%
2	QLFDGWO	**0.323**	**0.326**	**0.014**	683.93	100.00%
	GWO	0.356	0.369	0.020	695.80	100.00%
	MP-GWO	0.324	0.341	0.023	**666.29**	93.33%
	PSO-GWO	0.393	0.393	0.000	695.73	76.67%
	WOA	0.325	0.337	0.024	722.52	83.33%
	PSO	0.326	0.338	0.020	712.61	66.67%
3	QLFDGWO	**0.357**	**0.369**	**0.003**	15.78	100.00%
	GWO	0.365	0.386	0.004	17.91	96.67%
	MP-GWO	0.368	0.403	0.004	17.38	100.00%
	PSO-GWO	0.368	0.374	0.022	**11.65**	96.67%
	WOA	0.382	0.409	0.007	17.85	93.33%
	PSO	0.372	0.402	0.012	16.41	96.67%
4	QLFDGWO	**0.768**	**0.793**	**0.012**	1431.52	93.33%
	GWO	0.805	0.883	0.022	**1173.81**	86.67%
	MP-GWO	0.893	0.981	0.058	1426.13	80.00%
	PSO-GWO	1.078	1.087	0.037	1521.33	86.67%
	WOA	0.777	0.842	0.020	1559.72	83.33%
	PSO	1.927	2.127	0.042	1358.94	76.67%
5	QLFDGWO	1.283	**1.319**	**0.014**	353.54	100.00%
	GWO	**1.279**	1.333	0.018	**328.67**	100.00%
	MP-GWO	1.282	1.336	0.023	362.53	96.67%
	PSO-GWO	1.332	1.349	0.021	348.44	96.67%
	WOA	1.308	1.332	0.016	332.28	93.33%
	PSO	1.411	1.427	0.025	366.90	96.67%
6	QLFDGWO	**0.273**	**0.298**	**0.012**	636.59	96.67%
	GWO	0.295	0.326	0.021	647.60	96.67%
	MP-GWO	0.281	0.322	0.028	701.37	90.00%
	PSO-GWO	0.292	0.331	0.030	674.55	83.33%
	WOA	0.359	0.381	0.019	**612.49**	96.67%
	PSO	0.300	0.334	0.037	628.66	93.33%

## Data Availability

Data will be made available on request.
